# 4116 Comprehensive training and support for Research Professionals at the University of Minnesota

**DOI:** 10.1017/cts.2020.204

**Published:** 2020-07-29

**Authors:** Jennifer Maas, Megan Hoffman, Jessica Wright

**Affiliations:** 1 University of Minnesota CTSI

## Abstract

OBJECTIVES/GOALS: Coordinating research studies is multifaceted and requires a foundational level of research knowledge, skills and abilities in order to contribute to high-quality, ethical research projects that adhere to local and federal regulations as well as Good Clinical Practice. Oftentimes, coordinators who are new to research or new to an institution have trouble navigating the research landscape. Departments within the University of Minnesota have limited resources to devote to developing robust training programs above and beyond protocol or department-specific training. Therefore, UMN’s CTSI created a comprehensive training and support program for research professionals at the University of Minnesota. METHODS/STUDY POPULATION: CTSI employs several strategies to provide a comprehensive training program for the University of Minnesota Research Workforce. The offerings are based on the The Joint Task Force for Clinical Trial Competency (JTF). In addition to training programs, valuable resources, materials, and connections are provided to trainees.

- An Onboarding process for new coordinators that includes a welcome email upon hire that provides resources as well an opportunity to meet face-to-face to get their questions answered about where to start with research training.
- Foundations for Research professionals, two week (20 hour) training program, provides a foundational level of knowledge to new coordinators via in-person and online training modules.
- Informed Consent 1 & 2 provides in-person training on the informed consent including the process, documentation, and ethical issues around consenting vulnerable populations.
- Over 40 on-line research training modules that coordinators can take at anytime.
- An active list serv that connects >600 research professionals with training updates and opportunities.
- Bi-weekly seminar series that provides a forum to share current regulations, best practices, resources, and guidelines pertaining to clinical research at the University.
- An online training “Roadmap” tool that customizes individual research training plans, and includes an inventory of training available.

RESULTS/ANTICIPATED RESULTS:

- 218 research professionals participated in our Foundations blended training program with 191 completing (88% completion rate) the entire training. A comprehensive assessment based on national competencies is completed by all participants at Baseline and Post training. Baseline scores average at 75% and Post scores average at 82% (7% increase). Satisfaction is measured and participants are overall satisfied with the training, 4 out of 5 on a Likert Scale.
- 353 research professionals have participated in our Informed Consent Session 1 & 2 in-person training. Satisfaction is measured and participants are overall satisfied with the training, 4.5 out of 5 on a Likert Scale.
- Over 190 research professionals have utilized our research on-line training modules.
- Training participants have been from 27 different departments across the University.
- The Clinical Research Professional Development Seminar Series has offered over 87 seminars with 4907 total attendees. These seminars are offered in-person and live stream.

DISCUSSION/SIGNIFICANCE OF IMPACT: Establishing a comprehensive training program at the University has streamlined the training that research professionals receive across departments. It also ensures that all coordinators have access to research training, a network of other research professionals, resources, and continuing education opportunities.

